# *SHOOT GRAVITROPISM 9* links sensory timing to the initial lateral root growth angle

**DOI:** 10.1073/pnas.2536276123

**Published:** 2026-07-22

**Authors:** Sophie Zoe Farkas, Federico Grippo, Denisa Oulehlová, Alberto González-Delgado, Seinab Noura, Sima Molazeinali, Krzysztof Wabnik, Matyáš Fendrych, Sascha Waidmann, Jürgen Kleine-Vehn

**Affiliations:** ^a^ Institute of Biology II, Chair of Molecular Plant Physiology, University of Freiburg, Freiburg 79104, Germany; ^b^ Center for Integrative Biological Signalling Studies, University of Freiburg, Freiburg 79104, Germany; ^c^ Department of Experimental Plant Biology, Faculty of Science, Charles University, Prague 128 00, Czechia; ^d^ Institute of Experimental Botany of the Czech Academy of Sciences, Prague 16502, Czech Republic; ^e^ Centro de Biotecnología y Genómica de Plantas, Universidad Politécnica de Madrid—Instituto Nacional de Investigación y Tecnología Agraria y Alimentaria (INIA), Consejo Superior de Investigaciones Científicas (CSIC), Pozuelo de Alarcón, Madrid 28223, Spain; ^f^ Department of Plant Biology, School of Biology, College of Science, University of Tehran, Tehran 1417694411, Iran; ^g^ Future Forests Cluster of Excellence, University of Freiburg, Freiburg 79104, Germany

**Keywords:** root system architecture, auxin, amyloplast, lateral root angle

## Abstract

Plants strategically respond to gravity and actively set defined growth angles. These gravitropic set-point angles (GSAs) shape the width and depth of the root system, determining soil exploration for water and nutrient uptake. Despite its physiological importance, the concept by which lateral roots establish their growth angle has remained largely unclear. Here, we show that temporal regulation of gravity responsiveness within a defined developmental phase sets the initial GSA in lateral roots. Stage-specific transcriptome profiling revealed that *SHOOT GRAVITROPISM 9* (*SGR9*) is upregulated in emerged lateral roots as they first respond to gravity. Loss of SGR9 slows amyloplast sedimentation and delays the PIN3-dependent establishment of asymmetric auxin distribution. Notably, the delay in the gravity response alters, rather than merely postpones, the initial stable growth angle. Our findings reveal that a developmental time window enables transient sensory dynamics to be translated into stable initial root growth angles, illustrating how developmental timing can convert short-lived signals into lasting form.

## Significance

The angles at which roots grow into the soil determine how plants search for water and nutrients and are central to plant performance and stress resilience. We identify *SHOOT GRAVITROPISM 9* as a regulator of gravity perception in lateral roots, thereby uncovering that the establishment of the initial root growth angles is defined within a developmental time window. This conceptual framework illustrates how plants convert transient sensory experiences into architectural traits through temporally gated competence phases.

The angular growth of secondary roots is a key determinant of the root system architecture, influencing how plants explore the soil and access water and nutrients (1, 2). Upon emergence from the primary root, the stage I lateral roots initially grow at a near-perpendicular angle (3). Their gravity-sensing columella cells then mature, enabling starch-filled plastids (amyloplasts/statoliths) to sediment and provide a physical cue for gravity perception (3–5). This process provides directional information for the polarization of PIN3 auxin transporters, driving asymmetric auxin flow and gravitropic bending of stage II lateral roots (3, 6–8). However, unlike the primary root, stage II lateral roots display only a partial gravitropic response, establishing a defined angle early after emergence. This angular growth is maintained in stage III lateral roots and promotes radial soil colonization (3, 9). The characteristic angle at which plant organs grow relative to gravity is called the gravitropic set-point angle (GSA). Accordingly, we define the first stable growth angle that a lateral root develops hereafter as the initial GSA. Across diverse species, this initial GSA of lateral roots is quantitatively defined and kept for a substantial developmental period, underscoring its contribution to root traits. While the establishment of the initial GSA of lateral roots is tightly regulated in stage II and remains well-defined at stage III, the lateral root GSA becomes highly variable at stage IV (3, 7, 10). Accordingly, the establishment and maintenance of this initial GSA is of high ecological and agronomic importance. The underlying mechanism that defines this first stable growth angle in lateral roots is currently poorly understood. PIN proteins are temporally downregulated in stage III lateral roots, explaining why these lateral roots maintain the initial growth direction (3). Subsequently, the repression of columella PIN proteins is released (3, 7), and lateral root angles are stabilized through feedback between gravitropic and antigravitropic input signaling (6, 8, 9). However, how the specific initial growth angle conceptually emerges de novo in stage II lateral roots remains an open question. Here, we show that gating of a transient sensatory event gives rise to the initial GSA.

## Results and Discussion

In this study, we aim to investigate the establishment of the initial GSA in freshly emerged lateral roots. To tackle this eminent research question, we envisioned to genetically induce a delay in the actual perception of, and consequently the response to, gravity in stage II lateral roots. If angle control is mechanistically predetermined, such a delay should only postpone the formation of this initial angle but would not alter GSA control.

**Fig. 1.**
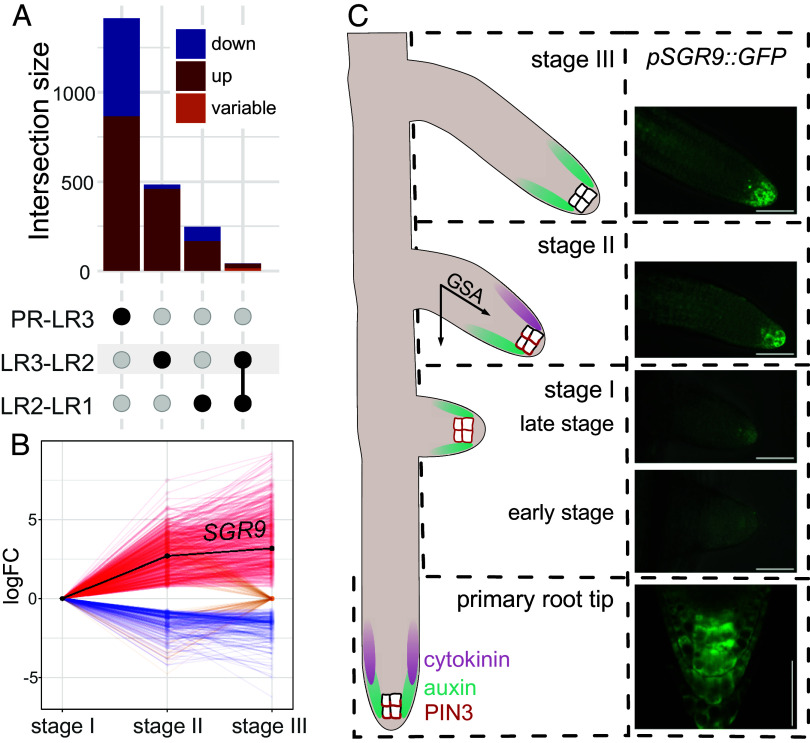
We performed stage-dependent dissection of lateral roots and RNA sequencing to find candidate genes that are involved in the stage I to stage II transition. (*A*) The RNA-Seq results show the DEGs in different stages (Dataset S1). We compared gene expressions based on developmental stages, such as comparing the primary root to stage III (PR-LR3), stage III to stage II (LR3-LR2), and stage II to stage I (LR2-LR1). Genes that were consistently upregulated or downregulated across multiple comparisons were labeled (red and blue, respectively), while genes with inconsistent regulation across comparisons were considered variable and depicted in orange. (*B*) Results of the RNA-Seq showing the gene expression patterns in lateral roots comparing stage I to stage II and stage III. Color coding is the same as in Fig. 1*A*, genes that are upregulated are red, downregulated genes are blue, variable genes are orange, and *SGR9* is highlighted in black. (*C*) Graphical representation of the different lateral root stages and the primary root on the left with corresponding images of *pSGR9::GFP* on the *Right*. (Scale bar, 50 µm.)

To identify molecular components that define the dynamics of gravity perception in emerged lateral roots, we initially performed a lateral root organ- and stage-specific RNA sequencing by dissecting stage I (LR1), II (LR2), and III (LR3) lateral roots as well as primary root tips (PR) for comparison. We noted that the lateral root transcriptome was highly distinct from the main root, showing 1.446 Differentially Expressed Genes (DEGs) between primary root and stage III lateral roots (Fig. 1*A* and Dataset S1). This illustrates a strong deviation in identity between main roots and young lateral roots. Moreover, comparative analysis revealed 299 DEGs between stage I and stage II, and 560 DEGs between stage II and stage III, highlighting progressive transcriptional changes during the earliest stages of lateral root maturation (Fig. 1 *A* and *B* and Dataset S1). Notably, most DEGs were unique to each developmental transition (LR1-3), with only 45 genes shared between the stage I vs. stage II and stage II vs. stage III comparisons, suggesting distinct transcriptional programs at each stage of lateral root development (Fig. 1*A* and Dataset S1). To uncover regulators of gravity perception, we focused on transcript dynamics between stage I and stage II lateral roots (Fig. 1*A*), which mark developmental maturation from nongravitropic to gravitropic lateral roots (3). This yielded into the identification of several gene candidates that could play a role in the establishment of the initial GSA in stage II lateral roots.

While this organ- and stage-specific approach opens the opportunity to identify novel regulators, we focused in this study on known components with a potential unknown role in defining gravitropic competence in lateral roots. Using this strategy, we identified *SGR9* as a candidate gene, showing increased expression as emerged lateral roots transitioned to become responsive to gravity (Fig. 1 *B* and *C*). In shoots, SGR9 promotes amyloplast sedimentation by modulating their interactions with the actin cytoskeleton, thereby fine-tuning the gravitropic response (11). Although its function in lateral roots is largely unknown, the established role of SGR9 in promoting amyloplast sedimentation in shoots made it a compelling candidate to test for a similar function in emerged lateral roots.

To further assess the role of *SGR9* in lateral roots, we first determined its spatial expression pattern, using the *pSGR9::GUS* and *pSGR9::GFP* transgenic lines (11). We detected *SGR9* expression in gravity-sensing columella cells of both lateral and primary roots (Fig. 1*C*). In agreement with our transcriptome profiling, we detected low *SGR9* expression in the columella cells of older emerged stage I lateral roots, which strongly increased in stage II and stage III lateral roots (Fig. 1*C*). Based on this spatiotemporal expression analysis, we conclude that SGR9 could function in the gravity-sensing cells of emerged lateral roots.

**Fig. 2.**
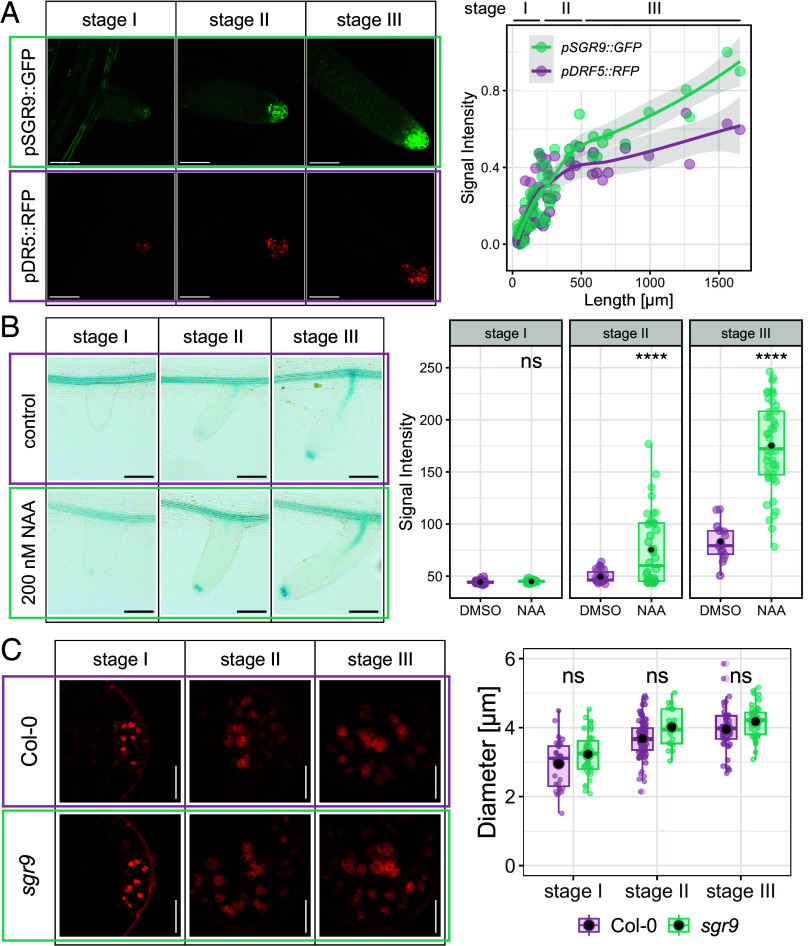
Expression analysis of *SGR9* in lateral roots. (*A*) The expression of *SGR9* (*pSGR9::GFP*) increases during development from stage I to stage III lateral roots. The increasing *SGR9* expression positively correlates with the increasing auxin signaling (based on the *pDR5::RFP* auxin reporter line) in the lateral root tip. (Scale bar, 50 µm.) Sample number is n*_pSGR9::GFP×DR5::RFP_* = 55. Values were normalized in the range of 0 to 1. (*B*) Upon exogenous auxin application (200 nM NAA), *SGR9* expression is higher in stage II and stage III lateral root tips compared to DMSO control. (Scale bar, 100 µm.) Sample numbers are n*_DMSO, stage I_* = 30, n*_DMSO, stage II_* = 26, n*_DMSO, stage III_* = 14, n*_NAA, stage I_* = 38, n*_NAA, stage II_* = 40, n*_NAA, stage III_* = 47. For statistical analysis *t* test was performed, *P*-values are p*_stage I_* = 0.09 (ns), p*_stage II_* = 7.6 × 10^−05^ (****), p*_stage III_* = 6.4 × 10^−16^ (****), for each stage NAA treatment was compared to DMSO control. (*C*) We used propidium iodide starch staining to examine the potential effect of the *sgr9* mutation on amyloplast size. We did not observe a significant difference in amyloplast size in stage I–III lateral roots of *sgr9* compared to WT. (Scale bar, 10 µm.) Sample numbers are for Col-0 n_*stage I*_ = 31, n_*stage II*_ = 82, n_*stage III*_ = 45, and for *sgr9* n_*stage I*_ = 39, n_*stage II*_ = 17, n_*stage III*_ = 42. For statistical analysis *t* test was performed, *P*-values are p*_stage I_* = 0.093 (ns), p*_stage II_* = 0.051 (ns), p*_stage III_* = 0.068 (ns), for each stage *sgr9* was compared to Col-0 control. (*B* and *C*) Boxplots show the first quartile, the median, the third quartile, and the mean value is depicted with a black circle.

We previously reported that nuclear auxin responses in columella cells exhibit increased activity during the maturation of lateral roots (7). To explore a possible correlation between *SGR9* expression and nuclear auxin signaling, we crossed the synthetic *pDR5::RFP* nuclear auxin output signaling reporter with the *pSGR9::GFP* expression reporter. Throughout lateral root development (stages I–III), increasing *SGR9* expression closely correlated with the nuclear auxin signaling dynamics in the gravity-sensing columella cells (Fig. 2*A*). Both reporters showed a pronounced rise during stages I and II, marking the transition toward gravitropic growth. This is followed by an expression plateau of *SGR9* and nuclear auxin signaling reporters at stage III, when differential growth along the lateral root flanks ceases and lateral roots maintain their growth orientation (Fig. 2*A*). To directly test if auxin affects *SGR9* expression, we exposed *pSGR9::GUS* transcriptional reporter lines to exogenous application of phytohormones and examined GUS reporter activity. Treatment with the synthetic auxin 1-naphthaleneacetic acid (NAA, 200 nM, 24 h) significantly increased SGR9 expression in the columella cells of stage II lateral roots (Fig. 2*B*). This effect was restricted to columella cells of stage II and III, whereas columella cells of early stage I lateral roots were largely unaffected (Fig. 2*B*). Conversely, blocking auxin transport pharmacologically with N-1-naphthylphthalamic acid (NPA) decreased SGR9 expression in columella cells of emerged lateral roots (*SI Appendix*, Fig. S1*A*), proposing that endogenous auxin contributes to setting *SGR9* expression in gravicompetent cells. Notably, cytokinin treatment (6-benzylaminopurine, BAP, 200 nM, 24 h) had no detectable effect on *SGR9* expression (*SI Appendix*, Fig. S1*A*), suggesting specificity to the observed auxin effect on *SGR9*. Accordingly, we assume that *SGR9* expression is controlled in an auxin-dependent manner, contributing to its stage-dependent expression in lateral root columella cells.

We also noted *SGR9* expression in the vasculature of main and lateral roots. The expression signal intensity in the lateral root vasculature base was significantly higher in auxin-treated stage III, but not stage I or II lateral roots (*SI Appendix*, Fig. S1*B*). Conversely, in the main root vasculature, the signal decreased upon auxin treatment in areas next to stage I and stage II but not stage III lateral roots (*SI Appendix*, Fig. S1*B*). While the function of SGR9 in vascular tissue is unknown, these data illustrate that *SGR9* shows tissue- and context-dependent auxin regulation, which may hint at an indirect mode of regulation. Using the RNA Sequencing (RNA-Seq) results, we grouped genes based on their expression dynamics along stage I–II and made a binding motif analysis of these groups (Dataset S2). We found that genes similarly regulated to *SGR9* have several potential binding sites for Teosinte branched1/Cycloidea/Proliferating cell factor (TCP) and WRKY transcription factors (*SI Appendix*, Fig. S1*C* and Dataset S2), which are known to be involved in auxin responses (12, 13). While this requires further investigation, it suggests that SGR9 is part of a developmental program contributing to lateral root maturation.

**Fig. 3.**
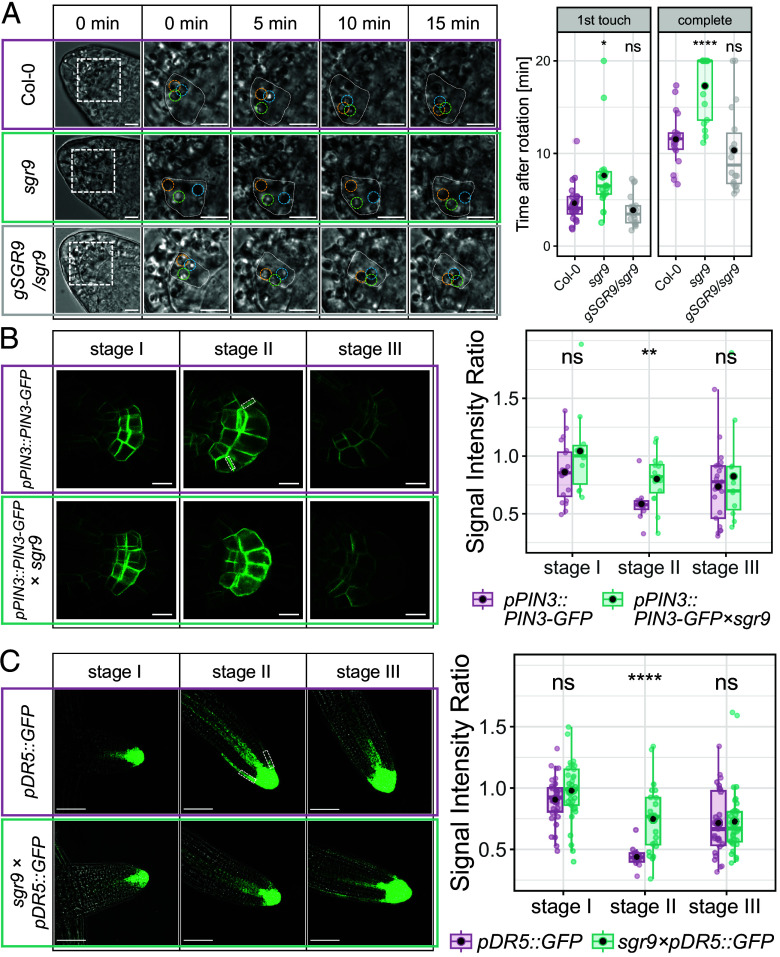
Amyloplast sedimentation, PIN3 polarization, and auxin signaling in the *sgr9* mutant line. (*A*) Amyloplasts sediment significantly slower in the *sgr9* mutant compared to WT (Col-0) or the complementation line (*gSGR9* in *sgr9*) after gravistimulation (180°) of stage III lateral roots. (Scale bar, 10 µm.) Sample numbers are n*_Col-0_* = 20, n*_sgr9_* = 17, n*_gSGR9/sgr9_* = 18. For statistical analysis *t* test was performed, *P*-values are for 1st touch p*_sgr9_* = 0.012 (*), p*_gSGR9/sgr9_* = 0.244 (ns), for complete sedimentation p*_sgr9_* = 6.3 × 10^−6^, p*_gSGR9/sgr9_* = 0.35 (ns), for each category genotypes were compared to Col-0 control. (*B*) To analyze PIN3 polarization in the lateral root columella cells, we used *pPIN3::PIN3-GFP* in Col-0 and *sgr9* backgrounds. We quantified the upper and lower membranes’ signal intensity, as shown on the *pPIN3::PIN3-GFP* stage II image. PIN3 has polar localization in stage II lateral roots in WT, however, this pattern is abolished in *sgr9*. (Scale bar, 10 µm.) Sample numbers are for *pPIN3::PIN3-GFP* n*_stage I_* = 20, n*_stage II_* = 11, n*_stage III_* = 21, and for *pPIN3::PIN3-GFP × sgr9* n*_stage I_* = 10, n*_stage II_* = 16, n*_stage III_* = 11. For statistical analysis *t* test was performed, *P*-values are p*_stage I_* = 0.20 (ns), p*_stage II_* = 0.005 (**), p*_stage III_* = 0.57 (ns), for each stage *pPIN3::PIN3-GFP × sgr9* was compared to *pPIN3::PIN3-GFP* control. (*C*) Auxin signaling measurement in the lateral roots is based on the signal intensity ratio between the upper and lower flanks, as shown on the *pDR5::GFP* stage II image. The signal intensity ratio differs significantly in the *sgr9* background (*sgr9 × pDR5::GFP*) in stage II lateral roots compared to WT (*pDR5::GFP*). (Scale bar, 50 µm.) Sample numbers are for *pDR5::GFP* n*_stage I_* = 32, n*_stage II_* = 11, n*_stage III_* = 27, and for *sgr9 × pDR5::GFP* n*_stage I_* = 33, n*_stage II_* = 25, n*_stage III_* = 34. For statistical analysis *t* test was performed, *P*-values are p*_stage I_* = 0.18 (ns), p*_stage II_* = 2.6 × 10^−5^ (****), p*_stage III_* = 0.88 (ns), for each stage *sgr9 × pDR5::GFP* was compared to *pDR5::GFP* control. (*A*–*C*) Boxplots show the first quartile, the median, the third quartile, and the mean value is depicted with a black circle.

The loss of *SGR9* function leads to disrupted amyloplast sedimentation in shoot endodermal cells (11), but its role in lateral roots is unknown. First, we examined whether the mutation of *sgr9* leads to changes in amyloplast size in stage I–III lateral roots. Using propidium iodide staining procedure, we did not observe differences in amyloplast size of *sgr9* mutant and wild type (WT) (Fig. 2*C*). Next, we aimed to assess whether SGR9 contributes to amyloplast sedimentation in lateral roots. In stages I to II, starch accumulation, and amyloplast sedimentation coincide and are hence technically challenging to quantify. Instead, we used time-lapse imaging of vertically oriented seedlings to investigate whether SGR9 controls the speed of amyloplast dynamics in already mature root columella cells. To assess sedimentation kinetics, individual stage III lateral roots were gravistimulated by rotating them 180°, and the root tip was imaged every 20 s for the duration of up to 20 min. We thereby were able to quantify the time duration for the first amyloplast sedimentation (1st touch) as well as the sedimentation of all amyloplasts (complete) (Fig. 3*A*). Amyloplast sedimentation in stage III lateral roots was not abolished but significantly delayed in the *sgr9* mutant when compared to the WT and the complementation line (Fig. 3*A*). Notably, we did not detect significant differences among these genotypes in amyloplast sedimentation in primary roots (*SI Appendix*, Fig. S2*A*). Taken together, this set of data proposes a role of SGR9 in contributing to amyloplast sedimentation in lateral roots. We hence assume the importance of SGR9 for the onset of the earliest gravity response in young, emerged lateral roots.

Amyloplast sedimentation initiates the relocalization of the LAZY proteins to the membrane of the new bottom side of the cell (4, 14), initiating the recruitment of RLD1 and subsequently the polarization of PIN3 proteins (15). Therewith, PIN3 will create an asymmetric auxin gradient at the bottom side of the root, triggering a differential growth response toward the gravity vector.

Accordingly, if SGR9 function contributes to amyloplast sedimentation dynamics in emerged lateral roots, the genetic interference with *SGR9* could quantitatively reduce PIN3 polarization in stage II lateral roots. To test this, we crossed functional *pPIN3::PIN3-GFP* reporter into the *sgr9* mutant background. To quantitatively address PIN3 polarization, we measured its signal intensity at the two flanks of columella cells (see outlined in Fig. 3*B*) and used its intensity ratio to depict PIN3 polarization. In agreement with previous reports (3), we observed a transient polarization of PIN3 in stage II columella cells (Fig. 3*B*). Compared to the WT, the polarization of PIN3 was significantly reduced in *sgr9* mutant in stage II lateral roots (Fig. 3*B*). This finding proposes that SGR9 contributes to amyloplast sedimentation and therewith PIN3 polarization toward the bottom side in stage II lateral root columella cells.

Gravity perception induces a preferential PIN3-dependent auxin transport toward the lower flank of stage II lateral roots. To quantify asymmetric auxin responses, we crossed *pDR5::GFP* auxin output reporters into *sgr9* mutant background. Both WT and *sgr9* lateral roots showed asymmetric *DR5* ratios between upper and lower flanks at stage II. However, the overall *DR5* asymmetry in *sgr9* was lower and more variable across individual roots compared to WT (Fig. 3*C*), indicating a delayed or less synchronized establishment of auxin asymmetry in stage II lateral roots. These findings demonstrate that SGR9 promotes amyloplast sedimentation and thereby contributes to the PIN3-dependent timing of gravitropic auxin responses at the lower root flank of stage II lateral roots.

Cytokinin provides an antigravitropic signal at the upper flank of the lateral roots, counterbalancing the auxin-driven gravitropic response (8). Notably, the asymmetry of cytokinin signaling depends on gravity-induced differential distribution of auxin (8). It remains, however, mechanistically unknown how auxin quantitatively and/or qualitatively shapes this asymmetric cytokinin response. Genetic crossing of the *pTCSn::GFP* cytokinin reporter into the *sgr9* background revealed cytokinin activity patterns that were quantitatively indistinguishable from WT, with no detectable loss of asymmetry (*SI Appendix*, Fig. S2*B*). This finding reveals that the weaker or delayed auxin redistribution in *sgr9* mutants remains sufficient to establish cytokinin asymmetry, consistent with a permissive or threshold-dependent mechanism.

**Fig. 4.**
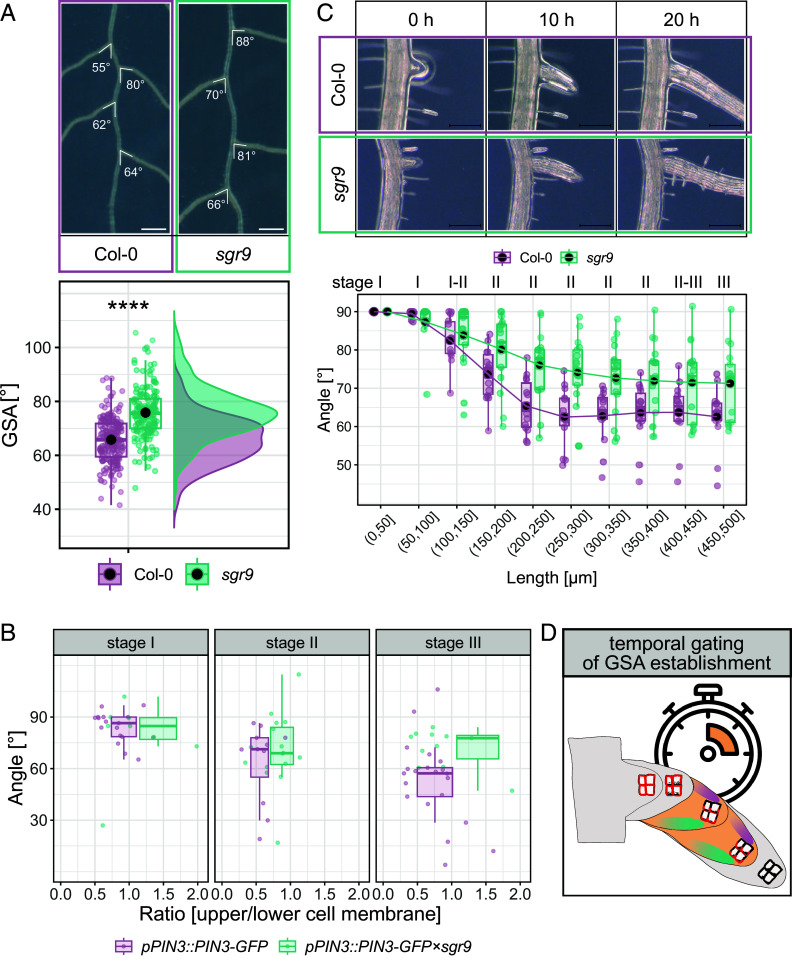
The assessment of the GSA of lateral roots in WT and *sgr9* mutant. (*A*) GSA of 9-d-old *sgr9* and Col-0 seedlings. (Scale bar, 1 mm.) Sample numbers are n*_Col-0_* = 176 and n*_sgr9_* = 164. For statistical analysis *t* test was performed, *P*-value is <2 × 10^−16^ (****), comparing *sgr9* to Col-0 control. (*B*) Angular growth of *pPIN3::PIN3-GFP* and *pPIN3::PIN3-GFP × sgr9* lateral roots (Fig. 3*B*). In the *sgr9* mutant, reduced PIN3 polarization in individual lateral roots leads to higher GSA, compared to control. Sample numbers are for *pPIN3::PIN3-GFP* n*_stage I_* = 17, n*_stage II_* = 11, n*_stage III_* = 21, and for *pPIN3::PIN3-GFP × sgr9* n*_stage I_* = 8, n*_stage II_* = 16, n*_stage III_* = 11. (*C*) Time series imaging with a vertical digital microscope to dissect the differences in the initial GSA over time in the *sgr9* mutant and in Col-0. (Scale bar, 200 µm.) Sample numbers are n*_Col-0_* = 14, n*_sgr9_* = 17. (*A*–*C*) Boxplots show the first quartile, the median, the third quartile, the half violin plots show the data distribution, and the mean value is depicted with a black circle. (*D*) Visual depiction of the temporal gating during GSA establishment.

The temporal delay in gravity perception in *sgr9* provides a genetic entry point to test whether the initial GSA is predefined. If this were the case, a delay in gravity response would be expected to postpone, but not alter, the establishment of the angle. We, hence, next examined whether *SGR9* contributes to the root system architecture establishment. Intriguingly, the lateral roots of *sgr9* mutants displayed a significantly increased GSA compared to WT (Fig. 4*A*). In agreement, the relative reduction in PIN3 polarization in individual *sgr9* mutant lateral roots corresponded with the reduction in angular growth across stages I–III (Fig. 4*B*). This set of data suggests that a *SGR9*-reliant delay in gravitropism is capable to alter the initial GSA in stage II lateral roots.

Previous work has shown that SGR9-dependent amyloplast sedimentation in shoots is linked to the actin cytoskeleton (11). Actin also contributes to gravitropism in primary roots by influencing amyloplast sedimentation, affecting endoplasmic reticulum functions, and PIN repolarization through vesicle trafficking (16). To test whether disrupting actin function can also set a new GSA, we interfered with actin genetically and pharmacologically. The *act8* mutant, as well as Latrunculin B treatments, induced a higher GSA than compared to controls (*SI Appendix*, Fig. S2*C*). Notably, Latrunculin B did not modulate the GSA of *sgr9* mutants (*SI Appendix*, Fig. S2*D*), suggesting that SGR9 may also intersect with the actin cytoskeleton during gravitropic responses in lateral roots. Moreover, it proposes that limiting gravitropic competence during stage II does not merely delay but leads to the establishment of a distinct initial GSA in emerged lateral roots.

To test if the observed effects of *SGR9* are specific to the GSA of lateral roots, we next examined gravitropic competence in main roots. In contrast to the altered initial GSA in lateral roots, the gravitropic response of the primary root was unaffected in *sgr9* mutants (*SI Appendix*, Fig. S3 *A* and *B*). This reveals that *sgr9* mutants are not generally defective in gravitropic root growth and hence proposes a specific function of *SGR9* in establishing the initial GSA in lateral roots.

Our data propose that a delay in gravity perception changes the initial GSA in stage II lateral roots, which may indicate that its establishment is temporally gated. We previously revealed that PIN3 is after GSA establishment transiently repressed in stage III lateral roots (3). We, hence, also tested whether *SGR9* contributes to the timing of PIN3 repression in stage III lateral roots (3). Notably, PIN3 repression in columella cells was indistinguishable in both WT and *sgr9* mutants (*SI Appendix*, Fig. S3*C*). These results suggest that *SGR9* primarily affects early polarization dynamics rather than later PIN3 repression.

To validate whether SGR9 contributes to establishing a stable GSA in lateral roots or whether lateral roots are merely defective in gravitropism, we reoriented seedlings and monitored lateral root gravitropism. The *sgr9* mutant stage III/III+ lateral roots were fully responsive to gravity stimulation and reestablished angles relative to the gravitropic vector, which were close to their original GSA (*SI Appendix*, Fig. S3*D*). Because lateral roots grow obliquely on both sides of the main root, the gravity response involves distinct bending on both sides of the main root, leading to gravity-induced downward bending on the upper/right side, and upward bending on the lower/left side (6), which was both intact in *sgr9* mutants (*SI Appendix*, Fig. S3*D*). This set of data reveals that SGR9 is a bona fide regulator of GSA in lateral roots.

To assess whether SGR9 specifically affects initial GSA establishment in stage II or also influences later stages of lateral root development, we examined the duration of stage III lateral roots maintaining a stable growth angle before they transit to stage IV by resuming bending toward gravity and altering their GSA (7). This is also referred to as the plateau phase, as it is characterized by stable GSA maintenance. Following previous methodology (7), we quantified the length of the plateau phase and found no significant difference between *sgr9* mutants and WT (*SI Appendix*, Fig. S3*E*). This reveals that SGR9 does not measurably affect the timing of later gravitropic behaviors in lateral roots, supporting a specific role in establishing the initial GSA during a defined developmental window.

We next used vertically oriented time-lapse imaging to directly visualize freshly emerged lateral roots establishing and stabilizing their growth angles over time. This analysis revealed that both WT and *sgr9* mutant lateral roots, despite setting distinct angles, completed their angle-setting phase within a similar time frame (Fig. 4*C*). Our findings, hence, collectively demonstrate that SGR9 does not modulate the duration of the angle-setting phase but the timing of gravity response within it.

Our set of data indicates that SGR9 contributes specifically to GSA establishment during a defined developmental phase, rather than broadly impairing gravitropic responsiveness. Accordingly, *sgr9* mutants would be expected to remain responsive to internal and external signals that modulate GSA. Consistent with this, auxin and cytokinin treatments induced smaller and larger GSA values, respectively, in both WT and *sgr9* mutants (*SI Appendix*, Fig. S4 *A* and *B*). To complement the hormonal treatments, we next examined the effects of altered ambient temperature. We observed an increased initial GSA at 29 °C and a decreased initial GSA at 12 °C in both WT and *sgr9* mutants (*SI Appendix*, Fig. S4*C*). These results indicate that SGR9 contributes to GSA establishment by controlling the timing of gravitropic responses, rather than their overall responsiveness.

Our results identify the timing of sensory activation as a determinant of initial growth direction in lateral roots, revealing a temporal gating of GSA establishment in emerged lateral roots (Fig. 4*D*).

## Concluding Remarks

By providing a spatiotemporal transcriptomic map of lateral roots, we uncover a role for SGR9 in defining the GSA of emerged lateral roots by regulating the timing of amyloplast sedimentation in gravity-sensing columella cells. *SGR9* is specifically upregulated at stage II lateral roots, ensuring timely gravity perception and angle formation in this developmental stage. Loss of *SGR9* function consequently slows amyloplast sedimentation and delays PIN3-dependent formation of auxin asymmetry. Accordingly, *sgr9* mutants induce a delay in differential growth, leading to a shifted GSA, without impairing the overall gravitropic competence of lateral roots. These findings establish *SGR9* as a bona fide GSA regulator in freshly emerged stage II lateral roots. This work conceptually reveals that the initial growth angle of lateral roots is established during a defined developmental time window. Once this developmental phase lapses, the respective angle is kept as a GSA during the subsequent growth phase of stage III lateral roots.

Following the here described establishment of the initial GSA, the subsequent transient downregulation of PIN3 in columella cells in stage III lateral roots (3, 7) marks a phase of stable growth direction. This could establish a “neutral” configuration in which further gravity stimulation and thus response is minimized. At later developmental stages, renewed PIN expression and polarization dynamics in lateral root columella cells (3, 6, 7) in principle restore gravitropic responsiveness. Here, previous studies have established that PIN polarization in lateral root columella cells is dynamically regulated (6), consistent with an active gravitropic and antigravitropic feedback system that can correct deviations in growth direction.

We propose a coherent developmental framework in which these mechanisms act in sequence during development, forming a three-tiered system of GSA control: i) a transient developmental window in which gravity perception and response defines the initial growth angle, followed by ii) the transient downregulation of PIN3 in gravity-sensing columella cells to robustly keep this angular growth, and iii) a subsequent phase in which feedback mechanisms can dynamically maintain but eventually also alter this angular orientation. This sequence of developmental trajectories provides the basis for a robust angle formation as well as the capacity to acclimate root growth angle to changing developmental and environmental conditions.

The here uncovered principle highlights how a defined developmental competence window can translate transient sensory inputs to specify stable organ-level traits and opens potential avenues to optimize the root system architecture.

## Materials and Methods

### Plant Material and Growth Conditions.

In this study, *pDR5::GFP, pDR5::RFP, pPIN3::PIN3-GFP, pSGR9::GFP, pSGR9::GUS*, and *pTCSn::GFP* *Arabidopsis thaliana* seeds were used. Seeds of *sgr9* (SALK_070212) and *pSGR9::gSGR9-GFP/sgr9* complementation line were kindly provided by Miyo Terao Morita (11). Seeds were surface sterilized and stratified at 4 °C for 2 d in the dark. Seedlings were grown vertically on half Murashige and Skoog medium [½ MS salts (Duchefa), pH 5.9, 1% (w/v) sucrose (Duchefa), and 1% (w/v) agar (Duchefa)]. Plants were grown under long-day conditions (16 h light/8 h dark), 100 µmol photons m^−2^ s^−1^ light intensity, and 21 °C temperature.

For the amyloplast sedimentation experiments, seeds were surface sterilized by chlorine gas for 2 h (17). Seeds were sown on 1% (w/v) agar (Duchefa) with ½ Murashige and Skoog (MS), Duchefa, 1% (w/v) sucrose, adjusted to pH 5.8 with KOH 1 M, and stratified for 2 d at 4 °C. Seedlings were grown vertically in a growth chamber with 23 °C by day (16 h), 18 °C by night (8 h), 60% humidity, and light intensity of 120 µmol photons m^−2^ s^−1^.

### Chemicals and Treatments.

6-Benzylaminopurin (BAP, Sigma Aldrich), Latrunculin B (LatB, Biomol), 1-Naphthaleneacetic acid (NAA, Sigma Aldrich), and N-1-naphthylphthalamic acid (NPA, Duchefa) were dissolved in dimethyl sulfoxide (DMSO) (Duchefa). Temperature treatments were 12 °C for cold, 21 °C for control, and 29 °C for heat.

### RNA-Seq.

RNA extraction was done as described previously (8, 18). In brief, a pool of 10 LR or root tips was collected in 30 µL of 100% RNAlater (Thermo Fisher), and 500 µL of TRIzol (Sigma) was added, followed by brief vortexing (2× for 2 s each), and incubating at 60 °C for 30 min. 100 µL of chloroform was added, and then, the samples were vortexed briefly (2× for 2 s each) and incubated at room temperature for 3 min. After centrifugation at 12,000 × g for 15 min at 4 °C, the aqueous phase was transferred to a new tube. To precipitate the RNA, an equal volume of isopropanol and 1.5 µL of GlycoBlue (Thermo Fisher) were added followed by a −20 °C incubation for 15 to 18 h and centrifugation at >20,000 × g for 60 min at 4 °C. After removal of the supernatant, the pellet was washed by adding 500 µL of 75% ethanol, vortexing briefly and then centrifuged at >20,000 × g for 15 min at 4 °C. The 75% ethanol wash step was repeated 1×. As much ethanol as possible was removed, followed by the drying of the pellet by letting the Eppendorf tube sit on ice with the lid open for 10 min. Precipitated RNA was then resuspended with 5 to 12 µL of nuclease-free water, stored at −80 °C (8, 18). RNA samples were sent for sequencing to BGI China.

### Differential Gene Expression Analysis and Visualization.

RNA-Seq data processing and differential expression analysis were performed using the Galaxy platform (usegalaxy.eu). DEGs across developmental stages of lateral root development were identified from RNA-Seq data. Comparisons were performed between the primary root (PR) and the lateral root in stage 3 (LR3), as well as between consecutive stages (LR1 vs. LR2 and LR2 vs. LR3). DEGs were classified as up-regulated or down-regulated based on an adjusted *P*-value < 0.05 and an absolute log2 Fold-Change > 2; genes that did not fulfill these thresholds were considered not significantly regulated (Dataset S1). Consistency of regulation across comparisons was evaluated: Genes consistently upregulated or downregulated were labeled accordingly, whereas genes with inconsistent regulation were classified as variable. To assess the overlap and intersection of DEGs among comparisons, we used the UpSetR package (19) in R (version 4.5.1).

### Histochemical GUS Staining.

Treatments with NAA and BAP were performed on 8-d-old seedlings (transferred to supplemented media), and GUS staining was performed after 24 h of treatment. GUS histochemical staining of acetone-fixed 9-d-old seedlings containing *pSGR9::GUS* fusion constructs followed a previously described method (20) using 5-Bromo-4-chloro-1H-indol-3-yl β-D-glucopyranosiduronic acid (X-Gluc, Carl Roth) as substrate.

As described in ref. 8, seedlings were fixed in 90% acetone for 30 min. After washing with 0.1 M Na-phosphate buffer (pH 7), seedlings were incubated for 2 h at 37 °C in the GUS staining solution [2 mM X-Gluc (dissolved in DMSO), 0.1% Trition X-100, 10 mM EDTA, 0.5 mM potassium ferrocyanide, 0.5 mM ferrycyanide, 0.1 M Na-phosphate buffer pH 7]. Examination of stained seedlings and image acquisition were performed with a light microscope (Zeiss Observer Z1) equipped with a DFC 300 FX camera (Zeiss). The intensity of the staining was quantified as described (21) in a region of interest that was kept constant.

R programming was used to perform *t* test to evaluate the statistical significance of the differences observed between control and genotype/treatment.

### Microscopy.

Vertical confocal microscopy was performed using a ZEISS Vertical LSM 980 Axio Observer 7 Microscope. Fluorescence signals for GFP (excitation wavelength 488 nm, emission wavelength 509 nm) and RFP (excitation wavelength 590 nm, emission wavelength 612 nm) were detected with a 40× air objective. The fluorescence signal intensity (mean gray value) of the presented markers was quantified using the maximum projections obtained from the Z-stack series that were taken and analyzed using the ZEISS software ZEN 3.10.

For the amyloplast sedimentation experiments, imaging was performed using a vertical stage Carl Zeiss Axio Observer.7, equipped with Zeiss LD LCI Plan-Apochromat 40×/1.2 WI objective or Zeiss EC Plan-Neofluar 5×/0.16, the bright field signal was detected with the Orca Flash 4.0 V3 camera (2,048 × 2,048 px, 6.5 μm pixel size, Hamamatsu). Images were acquired using the Zen Blue Software (Zeiss).

The intensity of the signal was quantified in a region of interest that was kept constant. R programming was used to evaluate the statistical significance of the differences observed between control and genotype.

### Gravistimulation.

High-spatiotemporal analysis of gravitropic bending of the primary roots was scored in 5-d-old seedlings. To avoid mechanical pressure of the agar block, we used the “on agar” method, where the plants were transferred onto a thin ½ MS medium layer placed inside a custom 3D printed chambered cover glass. After 30 min (or longer) recovery, the plants were rotated 90° and imaged directly through the cover glass and the agar every 2 min for overall 60 min. Root bending angle was quantified using a semiautomated pipeline Gravifast by Nelson BC Serre (https://sourceforge.net/projects/gravifast/). Both methods are described in ref. 22. Next to that, full graviresponse of primary roots was analyzed by gravistimulating seedlings at 90° and imaging every 1 h for the duration of 6 h. Gravitropic bending of the lateral roots was performed via 30° gravistimulation of seedlings, as described in ref. 6, analyzing stage III/III+ lateral roots after 6 h of gravistimulation.

### Amyloplast Sedimentation.

We tracked amyloplast sedimentation in lateral roots of 7-d-old seedlings of the following genotypes: Col-0, *sgr9*, *pSGR9::gSGR9-GFP/sgr9* in a blinded setup. Seedlings were cut out from the plate together with a block of agar, placed in a microscopic chamber, and recovered for at least 30 min.

Amyloplast sedimentation in root tips of both primary and lateral roots was followed after a 180° rotation of the seedlings. Images were taken every 20 s for up to 20 min. The largest columella cell was picked and used for amyloplast movement quantification, where we focused on sedimentation times of the first amyloplast and all amyloplasts, respectively, reaching the new bottom.

### GSA Measurements.

Plates with 12 to 16-d-old seedlings were scanned, and the GSA of lateral roots (first 1 mm) was measured to the gravity vector (3) using ImageJ. Lateral roots were measured when the primary root was nearly straight. R programming was used to generate graphs of GSA distribution, *t* test was used to evaluate the statistical significance of the differences observed between control and genotype/treatment.

## Supplementary Material

Appendix 01 (PDF)

Dataset S01 (XLSX)

Dataset S02 (XLSX)

## Data Availability

Study data are included in the article and/or supporting information.
