# Supplementary material for: SHOOT GRAVITROPISM 9 links sensory timing to the initial lateral root growth angle
Source: Proc Natl Acad Sci U S A. 2026 Jul 22;123(30):e2536276123. doi: 10.1073/pnas.2536276123 (PMC13416789; doi:10.1073/pnas.2536276123)
Supplement: Supplementary file 1 — Appendix 01 (PDF) [file pnas.2536276123.sapp.pdf]

PNAS

Supporting Information for
SHOOT GRAVITROPISM 9 links sensory timing to the initial lateral root growth angle

Sophie Zoe Farkas, Federico Grippo, Denisa Oulehlová, Alberto González-Delgado, Seinab Noura, Sima Molazeinali, Krzysztof Wabnik, Matyáš Fendrych, Sascha Waidmann*, Jürgen Kleine-Vehn*

Sascha Waidmann, Jürgen Kleine-Vehn
Email: sascha.waidmann@icloud.com, juergen.kleine-vehn@biologie.uni-freiburg.de

This PDF file includes:

Figures S1 to S4
Legends for Datasets S1 to S2

Other supporting materials for this manuscript include the following:

Datasets S1 to S2

Figures

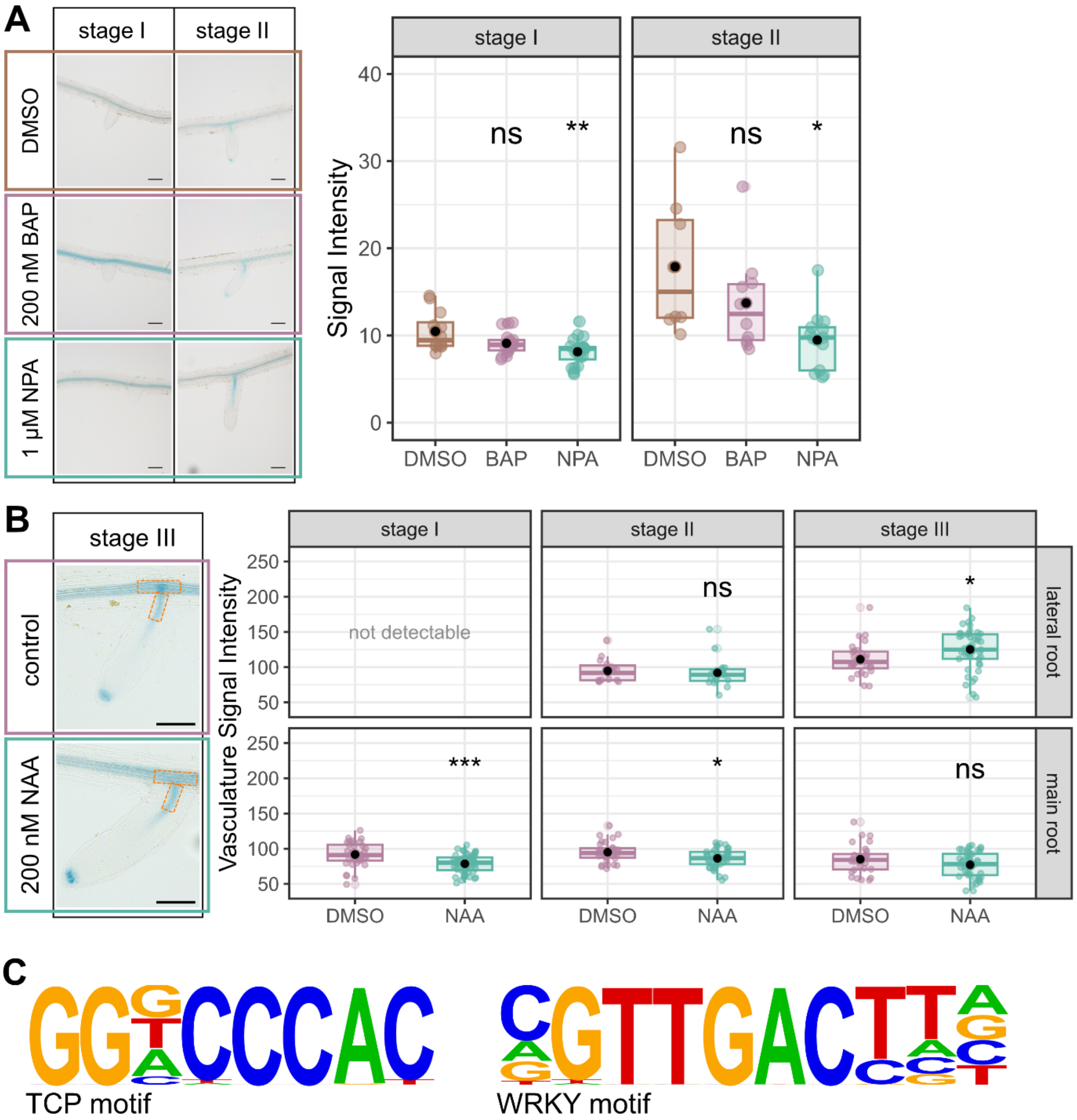

Fig. S1. ***(A)*** BAP and NPA treatment of *pSGR9::GUS*. Cytokinin treatment of *pSGR9::GUS*, with the synthetic cytokinin (200 nM BAP for 24 hours), did not significantly affect the expression level, but the inhibition of auxin transport (1 µM NPA for 24 hours) led to a lower *SGR9* expression, in stage I and stage II lateral root tips, compared to the DMSO control. Scale bars represent 100 µm. Sample numbers are $n_{DMSO,\ stage\ I}$ = 12, $n_{DMSO,\ stage\ II}$ = 8, $n_{BAP,\ stage\ I}$ = 17, $n_{BAP,\ stage\ II}$ = 10, $n_{NPA,\ stage\ I}$ = 20, $n_{NPA,\ stage\ II}$ = 13. For statistical analysis t-test was performed, p-values are $p_{BAP,\ stage\ I}$ = 0.0772 (ns), $p_{BAP,\ stage\ II}$ = 0.227 (ns), $p_{NPA,\ stage\ I}$ = 0.0056 (**), $p_{NPA,\ stage\ II}$ = 0.018 (*), for each stage BAP and NPA treatments were compared to DMSO control. ***(B)*** *SGR9* expression in the lateral and main root vasculature, region of interest is depicted with an orange rectangle. Scale bars represent 100 µm. Sample numbers are for the lateral root $n_{DMSO,\ stage\ II}$ = 15, $n_{DMSO,\ stage\ III}$ = 28, $n_{NAA,\ stage\ II}$ = 17, $n_{NAA,\ stage\ III}$ = 45, and for the main root $n_{DMSO,\ stage\ I}$ = 33, $n_{DMSO,\ stage\ II}$ = 31, $n_{DMSO,\ stage\ III}$ = 28, $n_{NAA,\ stage\ I}$ = 56, $n_{NAA,\ stage\ II}$ = 41, $n_{NAA,\ stage\ III}$ = 45. For statistical analysis t-test was performed, p-values are for the later root $p_{stage\ II}$ = 0.69 (ns), $p_{stage\ III}$ = 0.025 (*), and for the main root $p_{stage\ I}$ = 0.00056 (***), $p_{stage\ II}$ = 0.011 (*), $p_{stage\ III}$ = 0.11 (ns), for each stage and category NAA treatment was compared to DMSO control. ***(C)*** Potential TCP and WRKY transcription factor binding motifs (Dataset S2C, D). Binding motif analysis (Dataset S2A, B) was performed on genes similarly regulated as *SGR9* based on the RNA-Seq data (Dataset S1). ***(A, B)***

Boxplots show the first quartile, the median, the third quartile, and the mean value is depicted with a black circle.

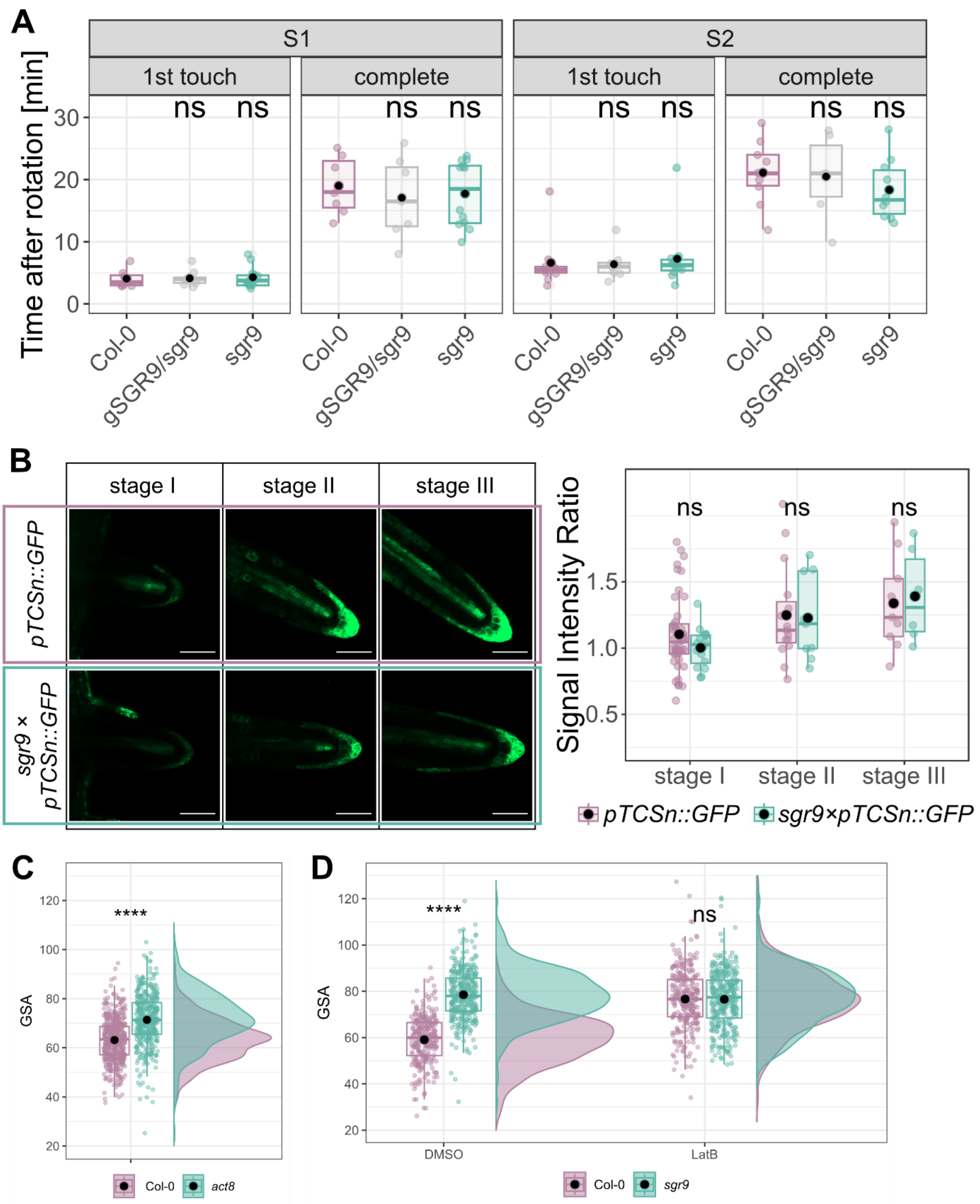

Fig. S2. ***(A)*** Amyloplast sedimentation in the first (S1) and second (S2) layer of the columella cells of the primary roots. Quantification is based on the 1st sedimented amyloplast and complete sedimentation of all amyloplasts to the new bottom of the cell. ***(B)*** Cytokinin signaling in the *sgr9* mutant line. Crossing the *pTCSn::GFP* cytokinin reporter line with the *sgr9* mutant showed us, that the mutation of this gene does not lead to a significant difference in the cytokinin signaling in stage I-II-III lateral roots. Sample numbers are $n_{pTCSN::GFP,\ stage\ I}$ = 44, $n_{pTCSn::GFP,\ stage\ II}$ = 15, $n_{pTCSn::GFP,\ stage\ III}$ = 9, $n_{sgr9\times pTCSn::GFP,\ stage\ I}$ = 16, $n_{sgr9\times pTCSn::GFP,\ stage\ II}$ = 9, $n_{sgr9\times pTCSn::GFP,\ stage\ III}$ = 6. For statistical analysis t-test was performed, p-values are $p_{stage\ I}$ = 0.082 (ns), $p_{stage\ II}$ = 0.781 (ns), $p_{stage\ III}$ = 0.784 (ns), for each stage *sgr9* × *pTCSn::GFP* was compared to *pTCSn::GFP* control. ***(C)*** Angular growth of lateral roots upon LatB treatment, inhibiting actin polymerization. GSA of Col-0 and *sgr9* seedlings treated with 100 nM LatB for 24 hours. Sample numbers are $n_{Col\text{-}0,\ DMSO}$ = 267, $n_{Col\text{-}0,\ LatB}$ = 294, $n_{sgr9,\ DMSO}$ = 339, and $n_{sgr9,\ LatB}$ =

336. For statistical analysis t-test was performed, p-values are $p_{DMSO} < 2\times10^{-16}$ (****), $p_{LatB} = 0.94$ (ns), for each treatment comparing *sgr9* to Col-0 control. ***(D)*** Angular growth of lateral roots in the *act8* mutant line, GSA of Col-0 and *act8* seedlings. Sample numbers are $n_{Col\text{-}0} = 517$ and $n_{sgr9} = 396$. For statistical analysis t-test was performed, p-value is $< 2\times10^{-16}$ (****), comparing *act8* to Col-0 control. ***(A, B, C, D)*** Boxplots show the first quartile, the median, the third quartile, and the mean value is depicted with a black circle.

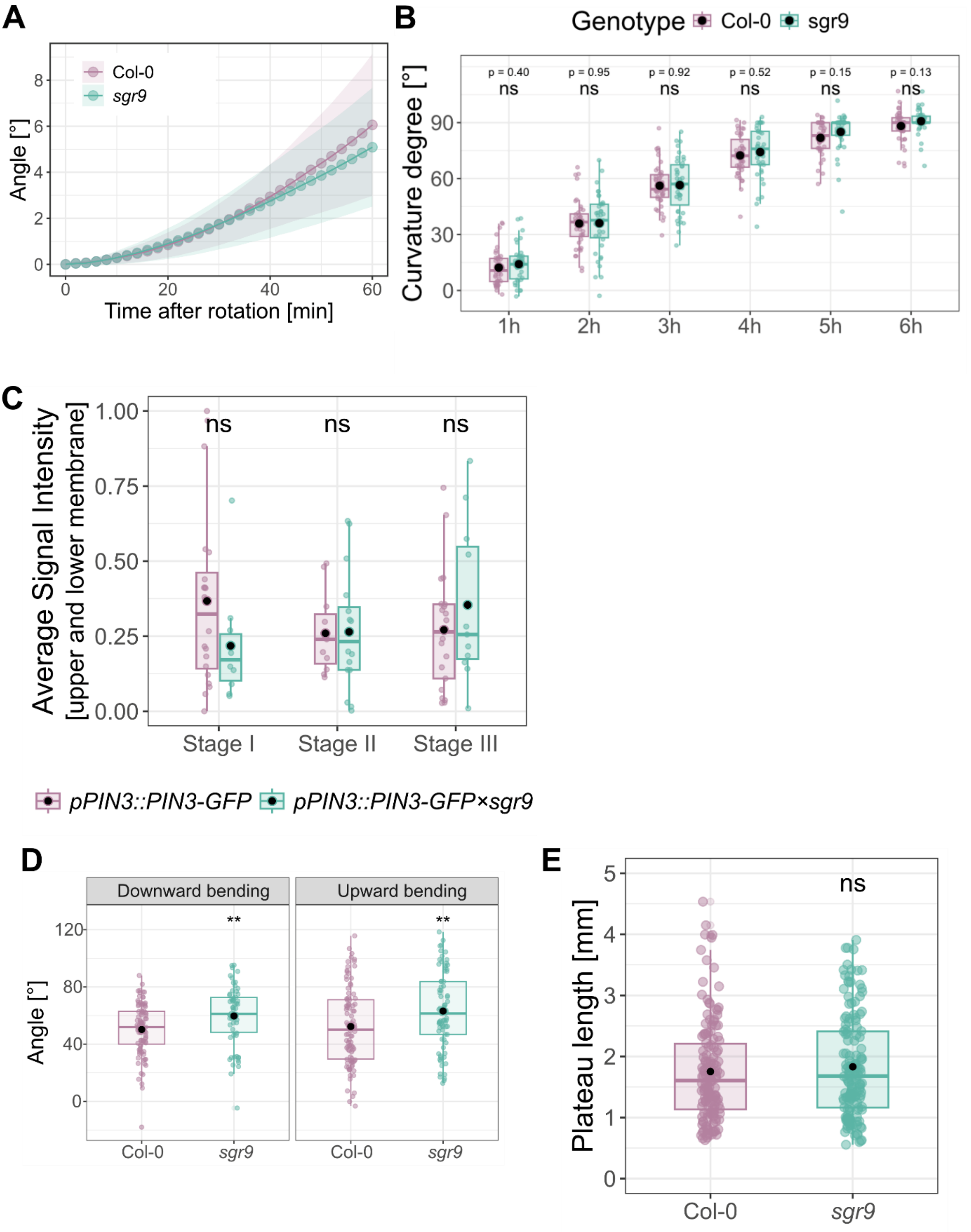

Fig. S3. ***(A)*** Graviresponse of the primary roots to 90° gravistimulation for 60 minutes, imaged every 2 minutes. Points depict the mean value, ribbon shows the standard deviation, sample numbers are $n_{Col\text{-}0}$ = 5, n_{sgr9} = 5. ***(B)*** Graviresponse of the primary roots to 90° gravistimulation for 6 hours, imaged every 1 hour. Sample numbers are $n_{Col\text{-}0}$ = 40, n_{sgr9} = 40. ***(C)*** *pPIN3::PIN3-GFP* signal disappearance in Col-0 and *sgr9* background. There was no significant difference in PIN3 signal intensity among stage I-III lateral roots, when comparing *pPIN3::PIN3-GFP×sgr9* to *pPIN3::PIN3-GFP*. Sample numbers are for *pPIN3::PIN3-GFP* $n_{stage\ I}$ = 26, $n_{stage\ II}$ = 14, $n_{stage\ III}$ = 22, and for *pPIN3::PIN3-GFP×sgr9* $n_{stage\ I}$ = 18, $n_{stage\ II}$ = 21, $n_{stage\ III}$ = 19. For statistical analysis t-test was performed, p-values are $p_{stage\ I}$ = 0.11 (ns), $p_{stage\ II}$ = 0.94 (ns), $p_{stage\ III}$ = 0.37 (ns), for each stage *pPIN3::PIN3-GFP×sgr9*

was compared to *pPIN3::PIN3-GFP* control. ***(D)*** Graviresponse of the lateral roots to 30° gravistimulation for 6 h. Sample number are for downward bending lateral roots $n_{Col\text{-}0}$ = 91, n_{sgr9} = 62, for upward bending lateral roots $n_{Col\text{-}0}$ = 94, n_{sgr9} = 78. For statistical analysis t-test was performed, p-values are $p_{downward\ bending}$ = 0.0047 (**), $p_{upward\ bending}$ = 0.0098 (**), for each category comparing *sgr9* to Col-0 control. ***(E)*** Plateau phase is the straight growth of stage III lateral roots before they start bending towards gravity in stage IV. We observed no significant difference when comparing this plateau phase length of the top 3 lateral roots of *sgr9* and Col-0 seedlings. Sample numbers are $n_{Col\text{-}0}$ = 144, n_{sgr9} = 144. For statistical analysis t-test was performed, p-value is p = 0.42 (ns) comparing *sgr9* to Col-0 control. ***(B, C, D, E)*** Boxplots show the first quartile, the median, the third quartile, and the mean value is depicted with a black circle.

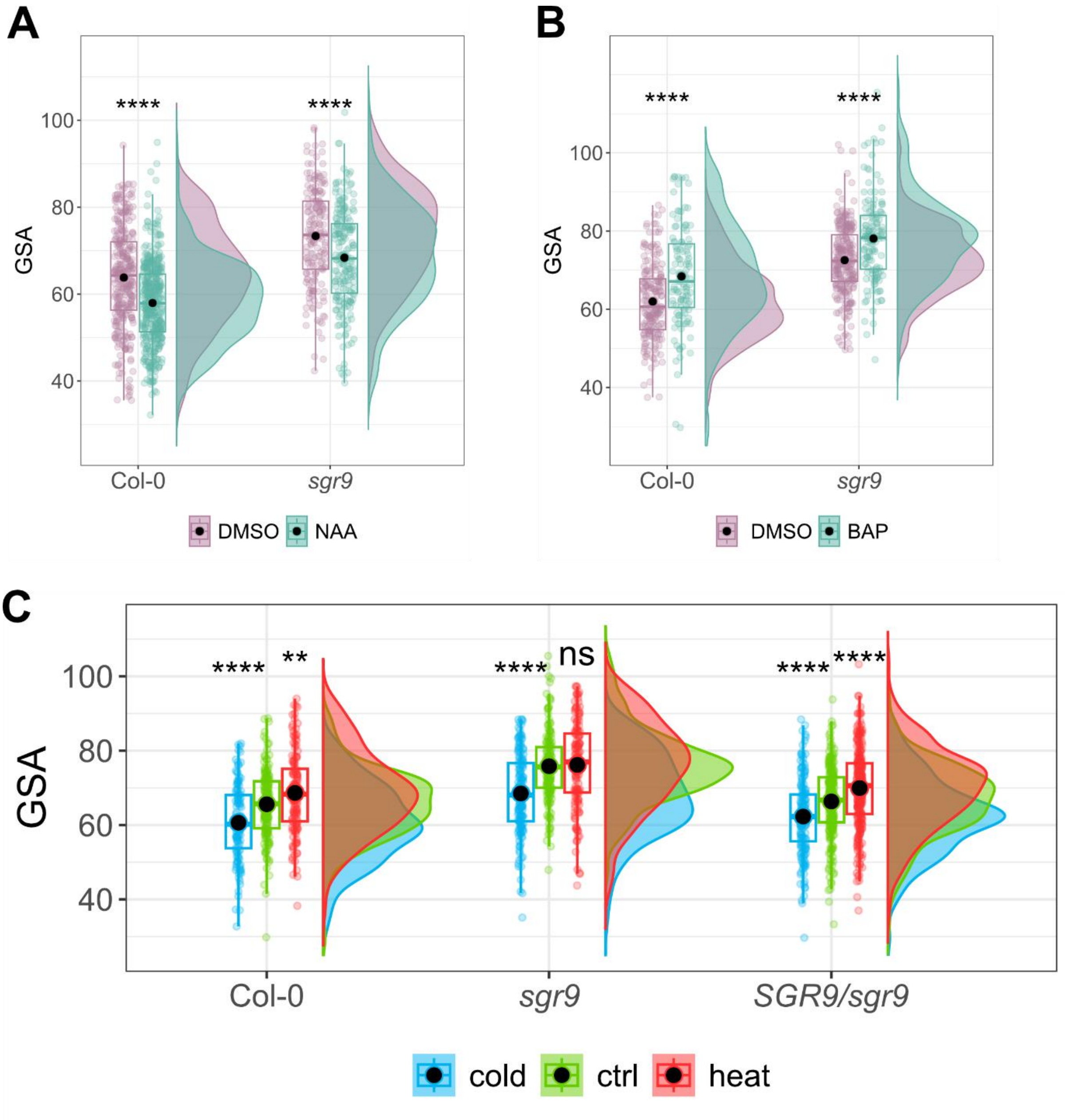

Fig. S4. ***(A)*** GSA of Col-0 and *sgr9* 7-day-old seedlings treated with 200 nM NAA for 5 days. Sample numbers are $n_{Col\text{-}0,\ DMSO}$ = 327, $n_{Col\text{-}0,\ NAA}$ = 429, $n_{sgr9,\ DMSO}$ = 183, and $n_{sgr9,\ NAA}$ = 198. For statistical analysis t-test was performed, p-values are $p_{Col\text{-}0} = 2.3\times10^{-13}$ (****), $p_{sgr9} = 2.2\times10^{-5}$ (****), for each genotype comparing NAA to DMSO control. ***(B)*** GSA of Col-0 and *sgr9* 7-day-old seedlings treated with 200 nM BAP for 4 days. Sample numbers are $n_{Col\text{-}0,\ DMSO}$ = 195, $n_{Col\text{-}0,\ BAP}$ = 116, $n_{sgr9,\ DMSO}$ = 255, and $n_{sgr9,\ BAP}$ = 140. For statistical analysis t-test was performed, p-values are $p_{Col\text{-}0} = 6.3\times10^{-6}$ (****), $p_{sgr9} = 6.9\times10^{-7}$ (****), for each genotype comparing BAP to DMSO control. ***(C)*** Ambient temperature changes have an effect on lateral root GSA, conditions were 12°C cold, 21°C control, 29°C heat. GSA of Col-0 and *sgr9* 7-day-old seedlings treated with different temperatures for 5-7 days. GSA decreases upon cold treatment and slightly increases upon heat treatment compared to control condition. There is a comparable shift in *sgr9* as in the Col-0 and *gSGR9/sgr9* complementation line. Sample numbers are for Col-0 n_{cold} = 124, n_{ctrl} = 176, n_{heat} = 138, for sgr9 n_{cold} = 153, n_{ctrl} = 164, n_{heat} = 144, for gSGR9/sgr9 n_{cold} = 174, n_{ctrl} = 230, n_{heat} = 278. For statistical analysis t-test was performed, p-values are for Col-0 $p_{cold} = 1.9\times10^{-5}$ (****), p_{heat} = 0.0087 (**), for *sgr9* $p_{cold} = 5.9\times10^{-10}$ (****), p_{heat} = 0.76 (ns), for *gSGR9/sgr9* $p_{cold} = 5.6\times10^{-5}$ (****), $p_{heat} = 7.6\times10^{-5}$ (****), compared to control conditions. ***(A, B, C)*** Boxplots show the first quartile, the median, the third quartile, the half violin plots show the data distribution, and the mean value is depicted with a black circle.

Dataset S1 (separate file). RNA-seq differential expression values.

Dataset S2 (separate file). ***(A)*** Motif enrichment analysis on genes similarly regulated as *SGR9*, all motifs. ***(B)*** Motif enrichment analysis on genes similarly regulated as *SGR9*, significant motifs. ***(C)*** Find Individual Motif Occurrences (FIMO) of TCP transcription factors on *SGR9*. ***(D)*** FIMO of WRKY transcription factors on *SGR9*.
